# Establishing General Working Population Norms for the Cognitive Symptom Checklist-Work

**DOI:** 10.1007/s10926-023-10104-8

**Published:** 2023-03-20

**Authors:** Johanna K. Ehrenstein, Saskia F. A. Duijts, Sander K. R. van Zon, Benjamin C. Amick, Sanne B. Schagen, Ute Bültmann

**Affiliations:** 1 Department of Health Sciences, Community and Occupational Medicine, University of Groningen, University Medical Center Groningen, Hanzeplein 1, PO Box 30.001, Groningen, 9700 RB, 9713 AV The Netherlands; 2 Division of Psychosocial Research and Epidemiology, The Netherlands Cancer Institute, Plesmanlaan 121, Amsterdam, 1066 CX The Netherlands; 3 Department of Public and Occupational Health, Amsterdam University Medical Centers (location Vrije Universiteit), De Boelelaan 1117, Van der Boechorststraat 7, Amsterdam, 1081 BT The Netherlands; 4 Amsterdam Public Health Research Institute, Societal Participation and Health, Amsterdam, The Netherlands; 5 Department of Research and Development, Netherlands Comprehensive Cancer Organisation (IKNL), Godebaldkwartier 419, Utrecht, 3511 DT The Netherlands; 6 Department of Epidemiology, Fay W. Boozman College of Public Health, University of Arkansas for Medical Sciences, 4301 West Markham Street, Little Rock, AR 72205 USA; 7 University of Arkansas for Medical Sciences, Winthrop P Rockefellor Cancer Institute, 449 Jack Stephens Dr, Little Rock, AR 72205 USA; 8 Department of Psychology, University of Amsterdam, Nieuwe Achtergracht 129-B, Amsterdam, 1018 WT The Netherlands

**Keywords:** Cognitive symptoms, Cancer, Self-report, Employment, Norms

## Abstract

*Purpose:* The Cognitive Symptom Checklist-Work (CSC-W) is a self-report measure to assess cognitive symptoms (i.e., memory and executive function) in working adults with cancer. To date, general working population norm data are lacking worldwide. We established CSC-W norm values in the general working population, and assessed associations of CSC-W scores with work and health-related factors. *Methods:* This cross-sectional study consisted of 1,000 Dutch working adults, of whom data was collected through an online respondent panel. The sample was stratified for sex and age, and data were weighted. Summary scores of the CSC-W total scale, and memory and executive function symptoms subscales, were determined (e.g., means, percentiles). Z- and T-scores were calculated, and analysis of (co)variance has been applied. *Results:* Cognitive symptom scores were relatively stable across age groups, but 18-39-year-old respondents reported lower memory and executive function than respondents in other age groups. Symptom scores of memory function (mean 29.1; SD = 16.7) were higher for all age groups and in both sexes compared to executive function (mean 22.1; SD = 16.8). No sex differences in memory and executive function were observed. Higher symptom scores were associated with performing non-manual work only, manual work only, self-reported long-term illness, and higher levels of depressive symptoms and fatigue. *Conclusion:* The CSC-W norms may enhance the interpretation and facilitate the analysis of self-reported cognitive symptoms in patients with cancer at work. Our findings may support health care professionals in identifying working adults with cancer with cognitive symptoms and in developing personalized treatment.

## Introduction

Working adults with cancer often have to cope with the effects of cancer and its treatment on physical, psychological and psychosocial health, and with limited understanding by their colleagues and employer [1, 2]. One of the most prevalent complaints in working cancer survivors is cognitive symptoms, which may persist for a significant period of time after return to work [3, 4]. Though cognitive symptoms affect cancer patients’ functioning at work [2], these symptoms are not systematically considered during and after a survivors’ return to work.

Cognitive symptoms among patients with cancer can be associated with the cancer itself [5, 6], cancer treatments and psychological consequences of cancer [3], and are measured via performance-based neuropsychological assessments [7] and self-report assessments [8]. While a neuropsychological test measures the cognitive capacity of an individual in a standardized environment that is independent of contextual factors, self-report measures of cognitive functioning, such as the Cognitive Symptom Checklist-Work Dutch Version (hereafter: CSC-W) [9], focus on the individual’s perception of their cognitive performance level in a work context. Patients with cancer with high levels of cognitive symptoms frequently report lower levels of quantity, quality, and timeliness of completed work, compared to those with low or no symptoms [10].

The CSC-W is a reliable and valid 19-item self-report measure of work-related cognitive symptoms in occupationally active adults with cancer [9]. The CSC-W is a modified version of the original English 21-item self-report measure, the Cognitive Symptom Checklist-Work-21 [11]. Dorland and colleagues (2016) showed that adults with cancer with higher CSC-W scores reported lower work functioning scores compared to those with lower CSC-W scores [9]. Further, the construct validity of the CSC-W is supported by positive correlations with fatigue and depressive symptoms [9].

To date, general working population norms for cognitive symptoms in working adults with cancer are lacking, but needed to interpret the prevalence levels of work-related cognitive complaints of patients with cancer compared to non-patients. Ultimately, normative data is needed by health care professionals to make informed treatment decisions. Further, an increased understanding of cognitive symptoms and associated factors may help health care professionals to identify patients at risk for cognitive symptoms. Therefore, this study aimed to develop general working population CSC-W norm scores to facilitate CSC-W interpretation among working adults with cancer, and to assess associations of CSC-W scores with work and health-related factors, known to be related to cognitive symptoms (i.e., type of work, long-term illness, depression, and fatigue). We hypothesised that adults with cancer with self-reported long-term illness, and higher levels of depressive symptoms and fatigue report higher levels of cognitive symptoms than those with no health problems, and low levels of depressive symptoms and fatigue.

## Methods

### Study Design and Population

We systematically collected data of participants aged 18–69 years from the general working population (n = 1,000) through Motivaction, a panel research company (https://www.motivaction.nl/panel-stempunt). Data were collected in December 2020, stratified for sex and age (18–39, 40–49, 50–59 to 60–69 years) with approximately 125 individuals per stratum. Ethical approval was granted by the non-Medical-Scientific Research with People Act (nWMO) applicable committee of the University Medical Center Groningen (METc 2020/343). Informed consent to participate was obtained from all participants prior to the study.

### Measures

*Cognitive Symptoms.* The CSC-W [9] (19 items, α = 0.96) was used to measure cognitive symptoms; it comprises two subscales to measure self-reported memory symptoms (8 items; α = 0.91) and executive function symptoms (11 items; α = 0.94). The memory symptoms subscale measures the frequency of symptoms experienced by aults with cancer with remembering. The executive function symptoms subscale measures the frequency of symptoms experienced by adults with cancer when using new information. All items are rated on a Likert-scale ranging from 0 (never) to 4 (always). The scale scores range from 0 to 100, with higher scores indicating a higher level of symptomatology or problems. In the current study, the total and subscale scores were obtained by summing the scores on each item, divided by the number of items. The average score was multiplied by 25. When 20% or more of the items were missing, the scale score was set to missing [9]. Missing data was classified only when participants indicated that an answer did not apply to them [9].

*Sociodemographic factors.* Sociodemographic factors included sex (female; male), age (in years), marital status (married/cohabitating; single/divorced), and level of education. Education was classified according to the definition of Statistics Netherlands (CBS) as: (1) low, i.e., primary, lower vocational, and lower secondary education; (2) medium, i.e., intermediate vocational and intermediate secondary, and (3) high, i.e., higher secondary, higher vocational, and university.

*Clinical factors and psychological symptoms.* Clinical factors included long-term illnesses or disabilities (e.g., high blood pressure; chronic pain; chronic respiratory diseases, such as asthma and chronic obstructive pulmonary disease) and medication use (i.e., none; psychiatric drugs; painkillers; sleeping pills; blood pressure-lowering drugs; other). Psychological symptoms included depressive symptoms and fatigue. Depressive symptoms were assessed using the self-report Patient Health Questionnaire-9 (PHQ-9; 9 items; α = .89) [12]. Response options range from 0 (not at all) to 3 (nearly every day). The maximum total score on this measure is 27, with higher scores indicating more severe depressive symptoms. The scores were dichotomized into ‘low’ (< 10) and ‘high’ (≥ 10, indicative of clinical depression levels) [12, 13]. Fatigue was assessed with the ‘fatigue severity’ subscale of the Checklist Individual Strength (CIS-8; 8 items; α = .82) [14]. The CIS-8 is a self-report instrument of prolonged fatigue in the working population. Response options range from 1 (yes, that is true) to 7 (no, that is not true). Total scores range from 8 to 56, with higher scores indicating more severe fatigue. A score greater than 35 indicates severe fatigue [3, 15]. Fatigue scores were dichotomized into (< 35) and ‘high’ (≥ 35).

*Work-related factors.* Work-related factors included the type of work (i.e., manual work only; non-manual work only; both manual and non-manual work) and psychosocial work environment factors. Psychosocial work environment factors included quantitative job demands (2 items; α = 0.57), work pace (2 items; α = 0.80), and job control (2 items; α = 0.66**)** measured with the Copenhagen Psychosocial Questionnaire (COPSOQ) [16]. Response options were assessed on a five-point scale (0 = never/hardly ever to 4 = always). Total scores ranged from 0 to 8, with higher scores indicating more quantitative job demands, higher work tempo, and low job control.

### Weighted Data

Some degree of underrepresentation of individuals with low educational attainment levels across all age groups was observed in our study sample. Therefore, the distribution on age, sex, and educational attainment level has been weighted according to the distribution from the gold standard 2020 from CBS [17] to ensure representativeness with the Dutch general working population [17].

### Statistical Analyses

Weighted and unweighted descriptive data analyses were performed to outline the baseline characteristics of the total study sample. Mean (M) scores and standard deviations (SD) of the CSC-W total scale and two subscales were determined by age group and sex. In addition to means, we also estimated median scores and the percentile distribution (the 5th, 25th, 75th, and 95th percentiles). We calculated norm-based scale Z-scores (mean of 0, SD of 1) and norm-based scale T-scores (mean of 10, SD of 50). Analyses of Variance were used to assess group differences between the strata. Analyses of Covariance were used to determine the associations between the CSC-W and work and health-related factors (i.e., type of work, long-term illness, depression, and fatigue). All analyses were adjusted for age, sex, and level of education. A two-sided alpha of 0.05 is used for significance. Statistical analyses were conducted using the Statistical Package for the Social Sciences (SPSS) version 26.

## Results

### Sample Characteristics

The mean age of the total weighted sample was 43.3 years (SD = 12.2), and 53.9% was male. 68% of the working adults were living with a partner (Table 1). Approximately 14.4% had a low educational level, while 44.9% had a high educational level. Half of the participants (54.9%) had a non-manual job, 25.6% had both manual and non-manual work, and 19.5% had a manual job. Respondents worked, on average, 33.9 (SD = 8.1, range = 12–80) hours per week. 39% of working adults reported having a doctor-diagnosed long-term illness. 7% of the participants had chronic respiratory diseases, such as asthma and chronic obstructive pulmonary disease (7.3%), followed by chronic pain (6.7%) and high blood pressure (6.5%). 16% of working adults in our sample reported more severe depressive symptoms, indicative of clinical depression. 25% of working adults experienced high levels of fatigue (see unweighted descriptives of the sample in Table 1).

**Table 1:** Sample characteristics (n = 1,000)

		Unweighted data	Weighted data
	*n*	Mean (SD) or %	Mean (SD) or %
*Sex*			
Females	503	50.3	46.1
Males	497	49.7	53.9
*Mean age (years)*	1000	48.2 (12.4)	43.3 (12.2)
*Age category*			
18 to 39	246	24.6	40.0
40 to 49	253	25.3	25.3
50 to 59	256	25.6	25.4
60 to 69	245	24.5	9.3
*Education*			
High	458	45.8	44.9
Medium	439	43.9	40.1
Low	99	9.9	14.4
Missing	4	0.4	0.6
*Marital status*			
Married/cohabitating	686	68.6	65.8
Single/divorced/separated	317	31.7	33.9
Prefer not to answer	3	0.3	0.3
*Cognitive symptoms total score*	968	23.8 (14.8)	25.1 (15.8)
*Memory symptoms*	982	28.0 (16.0)	29.1 (16.7)
*Executive function symptoms*	961	20.6 (15.7)	22.1 (16.8)
*Long-term illnesses or disabilities*			
None	557	55.7	60.5
High blood pressure	104	10.4	6.5
Other	101	10.1	8.5
Mental illness, such as depression, psychosis or anxiety disorder	60	6.0	6.3
Chronic pain	78	7.8	6.7
Chronic respiratory diseases such as asthma and chronic obstructive pulmonary disease (COPD)	72	7.2	7.3
Cardiovascular disease (including high blood pressure)	62	6.2	4.2
Diabetes mellitus	62	6.2	4.7
Mental illness, such as depression, psychosis or anxiety disorder	60	6.0	6.3
Autoimmune disease such as celiac disease, inflammatory bowel disease (IBD) / rheumatism / systemic Lupus erythematodes (SLE)	43	4.3	3.9
Thyroid disease	26	2.6	1.8
Cancer	24	2.4	1.9
Heart attack	12	1.2	1.0
Prefer not to answer	23	2.3	2.9
*Treatment*			
None	626	62.6	67.7
Other	144	14.4	8.5
Blood pressure-lowering drugs	140	14.0	9.5
Painkillers	82	8.2	8.2
Psychiatric drugs	48	4.8	5.0
Sleeping pills	21	2.1	2.7
Prefer not to answer	25	2.5	2.5
*Depressive symptoms*			
Low	877	87.7	84.5
High	123	12.3	15.5
*Fatigue*			
Low	762	76.2	75.5
High	238	23.8	24.5
*Type of job*			
Manual	176	17.6	19.5
Non-manual	564	56.4	54.9
Both manual and non-manual	260	26.0	25.6
*Psychosocial work environment*			
Quantitative job demands	1000	2.4 (1.7)	2.5 (1.7)
Tempo	1000	4.1 (1.8)	4.1 (1.8)
Job control	1000	4.5 (1.9)	4.4 (1.9)
*Contract hours*	1000	33.3 (8.3)	33.9 (8.1)
*Work Status*			
Self-employed	117	11.7	9.5
Employed	883	88.3	90.5
*Company size*			
< 10 employees	77	7.7	6.7
10 to 99 employees	217	21.7	25.3
100 to 499 employees	196	19.6	20.0
500 or more employees	434	43.4	41.6
Not applicable	62	6.2	5.1
Missing	12	1.2	1.2

Note: *SD*, standard deviation.

### CSC-W Total and Subscale Memory and Executive Function Scores

Working adults reported an average CSC-W total score of 25.1 (SD = 15.8) (Tables 2 and 3). Memory symptom scores (M = 29.1; SD = 16.1) were higher compared to executive function symptom scores (M = 22.1; SD = 16.8) (Tables 2 and 3**)**.

**Table 2:** CSC-W general working population normative data for women by cognitive symptoms total scores, and memory and executive function symptoms scores subscales stratified by age (weighted data)

	Total	Female				
		All female	18–39 yrs	40–49	50–59	60–69
***Cognitive symptoms*** ***total score***						
*M*	25.06	24.16	27.54	22.19	21.33	22.55
*SD*	15.85	14.12	14.84	13.34	13.00	13.38
*Percentile score*						
5	0.00	1.32	1.32	1.32	0.00	0.00
10	5.26	3.95	8.82	5.26	2.18	3.90
25	14.47	14.47	18.42	12.89	11.53	11.91
50	25.00	25.00	26.32	23.49	22.37	23.61
75	33.33	32.35	35.07	31.28	31.29	32.61
90	44.74	40.79	50.12	38.16	38.39	40.71
95	51.32	51.32	55.75	43.78	40.79	49.19
*Z-score*	0.00	0.05	0.35	0.01	-0.30	-0.19
*T-score*	50.00	50.50	53.50	50.10	47.00	48.40
***Memory symptoms*** ***subscale***						
*M*	28.98	28.65	32.09	26.75	25.45	27.65
*SD*	16.78	15.95	17.00	15.03	14.61	14.89
*Percentile score*						
5	0.00	0.00	3.13	0.00	0.00	0.00
10	6.25	6.25	9.38	6.25	3.13	6.18
25	18.75	18.75	21.88	15.63	15.63	15.63
50	28.13	28.13	31.25	25.58	25.00	28.13
75	40.63	37.50	40.63	37.50	34.38	40.63
90	50.00	50.00	56.25	43.75	46.88	46.88
95	56.25	56.25	62.50	50.00	50.00	51.04
*Z-score*	0.00	0.02	0.35	0.01	-0.30	-0.08
*T-score*	50.00	50.20	53.50	50.10	47.00	49.20
***Executive function symptoms subscale***						
*M*	22.21	20.87	24.20	18.88	18.32	18.76
*SD*	16.79	14.78	15.29	14.19	13.80	14.47
*Percentile score*						
5	0.00	0.00	0.00	0.00	0.00	0.00
10	0.00	0.00	2.27	0.00	0.00	0.00
25	9.09	9.09	15.91	6.82	4.55	5.91
50	22.73	22.50	25.00	18.18	18.18	16.80
75	31.82	29.55	30.80	27.40	29.55	27.78
90	43.18	38.64	47.94	36.36	34.48	38.64
95	50.00	50.00	57.69	38.64	40.90	49.53
*Z-score*	0	-0.03	0.26	-0.09	-0.18	-0.10
*T-score*	50	49.70	52.63	49.06	48.23	48.97

Note: *CSC-W*, Cognitive Symptom Checklist-Work, Dutch Version; *M*, mean; *SD*, standard deviation.

**Table 3:** CSC-W general working population normative data for men by cognitive symptoms total scores, and memory and executive function symptoms scores subscales stratified by age (weighted data)

	Total	Male				
		All male	18–39 yrs	40–49	50–59	60–69
***Cognitive symptoms*** ***total score***						
*M*	25.06	25.82	30.55	25.24	20.32	22.03
*SD*	15.85	17.14	18.69	17.81	12.59	13.55
*Percentile score*						
5	0.00	0.000	5.26	0.00	0.00	0.00
10	5.26	5.26	6.58	1.51	3.41	2.13
25	14.47	13.89	18.42	13.16	9.72	11.84
50	25.00	25.00	28.95	25.00	21.05	22.37
75	33.33	34.29	39.47	35.53	27.81	31.58
90	44.74	46.05	51.32	44.74	35.53	41.62
95	51.32	52.63	61.84	53.24	44.48	47.72
*Z-score*	0.00	0.05	0.35	0.01	-0.30	-0.19
*T-score*	50.00	50.50	53.50	50.10	47.00	48.40
***Memory symptoms*** ***subscale***						
*M*	28.98	29.25	33.91	28.47	23.75	26.28
*SD*	16.78	17.45	17.97	18.26	14.37	15.70
*Percentile score*						
5	0.00	0.00	7.61	0.00	0.00	0.00
10	6.25	9.38	12.50	3.58	5.50	4.57
25	18.75	17.98	21.88	17.46	13.21	14.60
50	28.13	28.13	34.38	28.13	21.88	28.13
75	40.63	40.63	43.75	40.63	34.38	36.43
90	50.00	50.00	54.61	50.00	43.75	49.68
95	56.25	59.38	59.75	57.53	50.00	53.56
*Z-score*	0.00	0.02	0.30	-0.03	-0.31	-0.16
*T-score*	50.00	50.20	53.00	49.70	46.90	49.40
***Executive function symptoms subscale***						
*M*	22.21	23.33	28.12	22.89	17.82	18.92
*SD*	16.79	18.24	20.26	18.54	13.44	14.07
*Percentile score*						
5	0.00	0.00	0.00	0.00	0.00	0.00
10	0.00	0.00	0.00	0.00	0.00	0.00
25	9.09	9.09	15.91	8.04	4.55	7.01
50	22.73	22.73	27.27	25.00	18.18	18.18
75	31.82	31.82	38.64	31.82	27.27	29.06
90	43.18	45.45	52.27	45.63	36.02	39.11
95	50.00	54.55	63.64	51.87	43.43	46.48
*Z-score*	0	0.03	0.39	0.10	-0.23	-0.13
*T-score*	50	50.30	53.90	50.96	47.71	48.72

Note: *CSC-W*, Cognitive Symptom Checklist-Work, Dutch Version. *M*, mean; *SD*, standard deviation.

### CSC-W Total and Subscale Memory and Executive Function Scores by Sex and Age

Cognitive symptoms total scores, and memory and executive function symptoms scores, were relatively stable across age groups, but 18-39-year-old respondents scored significantly higher in cognitive symptoms total and subscale scores compared to respondents in the other age groups (Tables 2 and 3**)**. No sex differences were found in cognitive symptoms total and subscale scores.

### Associations of CSC-W Scores with Work and Health-Related Factors

Respondents with a self-reported long-term illness or disability had higher CSC-W scores (indicating a higher level of cognitive symptoms at work) than those who reported no health problems (Table 4). Working adults who had a high depressive symptoms level and/or high level of fatigue reported higher CSC-W scores than those with a low level of depressive symptoms and/or fatigue. Working adults with a non-manual job reported higher CSC-W scores than those with both manual and non-manual work. Working adults with a manual job reported higher CSC-W scores than those with both manual and non-manual work.

**Table 4:** Associations of CSC-W scores with work and health-related factors (weighted data)

*Measures*	Cognitive symptoms total (CSC-W) M (95% CI)	F	p
*Morbidity* ^1,2,3^			
No long-term illness, or disability (*n* = 565)	21.16 (19.32-23.00)	59.46	< 0.001
Long-term illness or disability (n = 357)	29.18 (27.23–31.13)		
*Depressive symptoms (PHQ 9)* ^2,3^			
Low (n = 769)	22.39 (20.82-24.00)	151.28	< 0.001
High (n = 153)	38.49 (35.82–41.16)		
*Fatigue (CIS)* ^1,2,3^			
Low (n = 701)	23.18 (21.46–24.89)	29.41	< 0.001
High (n = 221)	29.62 (27.23–32.02)		
*Type of job* ^2,3^			
Manual (n = 176)	27.24 (24.52-30.00)	5.57	0.004
Non-manual (*n* = 502)	25.29 (23.34–27.24)		
Both manual and non-manual(n = 244)	22.27 (19.97–24.57)		

Significant covariates: ^1^ Sex ^2^Age Category ^3^ Education level

Note: *CSC-W*, Cognitive Symptom Checklist-Work, Dutch Version; *M*, mean; *CI*, confidence interval;

^a^ Post Hoc analyses showed significant differences between working adults with a non-manual and those with both manual and non-manual work and working adults with a manual job and those with both manual and non-manual work.

## Discussion

### Main Findings and Interpretation

The purpose of this study was to provide normative data from the general working population regarding cognitive symptoms, using the CSC-W. Cognitive symptoms scores were relatively stable across age and sex groups, suggesting overall norms may be used. Memory function symptom scores were higher for all age/sex groups compared to executive function. Higher symptoms scores were associated with performing non-manual work only, manual work only, self-reported long-term illness, and higher levels of depressive symptoms and fatigue.

Stable patterns of cognitive symptoms scores were observed across age groups, except for some consistently higher scores reported by 18–39-year-old respondents compared to respondents in the other age groups. Our results are similar to those reported in recent general population normative studies for the European Organization for Research and Treatment of Cancer Core Quality of Life Questionnaire (EORTC QLQ-C30). For instance, Nolte et al. (2019 and 2020) [18, 19], showed that for cognitive function, the youngest age group of 18–39 years scored lower/worse than any of the other age groups. This might be related to the accumulating demands in this life phase, including potential child care and career development. In previous research, it has also been shown that adults adjust health expectations with increasing age and that younger adults’ health perceptions are more influenced by health limitations than those of older adults [20].

The results of the current study also indicated that females and males do not rate their cognitive function at work differently. In earlier general population research, it has been shown that men tended to rate themselves lower (i.e., better) than women on the overall cognitive failure score, encompassing memory, attention, action, and perception, but these differences were negligibly small, and no differences were found in this specific study on the memory subscale [21].

Higher symptom scores were associated with performing non-manual work only, and manual work only. This is in line with previous research in working adults with cancer by Dorland and colleagues (2018) [3], which showed that adults with cancer with both manual and non-manual work report less cognitive symptoms over time, compared with adults with cancer with non-manual work only. The CSC-W total score was associated in expected ways with self-reported long-term illness, and with higher levels of depressive symptoms and fatigue. The findings of a systematic review suggested that cognitive symptoms among older adults are more common in those with a chronic condition compared to those who report no chronic illness [22]. Further, research in the general population already showed that depressive symptoms are associated with self-reported memory symptoms [23]. Previous research on the CSC-W in working adults with cancer similarly showed that CSC-W total scores were related to fatigue and depression among working adults with cancer [9].

### Strength and Limitations

A strength of our study was the use of an internet panel, which enabled access to a large and diverse sample. This resulted in no missing items for the CSC-W. Also, previous research showed that employing panel data for patient-reported outcomes is generally comparable to those of national norms [24]. Further, the sample is representative of the Dutch general working population due to the data collection and due to weighing the data, according to the distribution regarding age and sex, and educational distribution, from the gold standard 2020 from CBS [17]. A limitation of our study was that some degree of underrepresentation of individuals with low educational attainment levels across all age groups was observed. This difference was most pronounced in the oldest age group, where there were fewer participants with a low educational attainment level. Yet, this was expected, as it is common in the literature that individuals with low socioeconomic status are underrepresented in research [25]. To address this limitation, the distribution on age and sex, and educational attainment level has been weighted, as described.

### Implications for Practice and Research

The general working population CSC-W norms provide crucial information for health care professionals to enhance the interpretation and facilitate the analysis of self-reported cognitive symptoms in adults with cancer at work. CSC-W assessments can increase symptom awareness, help timely intervention, and can be used as a basis for communication. The interpretation of work-related self-reported cognitive symptoms, using CSC-W, depends on definitions of normal and abnormal, the context for the examination, the relationship to prior levels of function, and whether diagnostic and therapeutic interventions are implied and anticipated. As with all patient-reported outcome data, for which reference standards are available, clinical judgment is required to weigh the possibilities of error in the individual test score and consider explanations of results substantially higher than expected values. A comprehensive way for a healthcare professional to use these results is to locate an individual patient’s CSC-W score within the percentile distribution shown for that patient’s sex and age. In case of higher cognitive symptom scores, the health care professional could, for example, consider referral to a neuropsychologist for an objective assessment of neuropsychological functioning. Specific work-related support could also be arranged, such as individual guidance, psycho-education, cognitive strategy training, and/or fatigue management (e.g., the Internet-based cognitive rehabilitation for WORking Cancer survivors (i-WORC) [26]. Potential work accommodations for adults with cancer, who experience cognitive symptoms, may depend on the job type and include working fewer hours per week, with an adjusted work schedule, adapting work tasks, and changing the workplace (i.e., own office with less distraction).

In future studies, normative data should be established in other countries to account for different work contexts, labor markets, and social security systems. Further, these normative data should be applied to other groups, with expected differences in cognitive symptom scores. Also, establishing cut-off points for the CSC-W would further facilitate interpretation of its scores.

## Conclusion

In this study, general working population normative data for cognitive symptoms for use in working adults with cancer on the CSC-W were established. The results provide a valuable resource for anyone assessing and detecting cognitive symptoms in working adults with cancer.

## Data Availability

The data that support the findings of this study are not openly available.

## List of Abbreviations

CIS-8: Checklist Individual Strength
CSC-W: Cognitive Symptom Checklist-Work Dutch Version
COPSOQ: Copenhagen Psychosocial Questionnaire
EORTC QLQ-C30: European Organization for Research and Treatment of Cancer Core Quality of Life Questionnaire
M: Mean
nWMO: Non-Medical-Scientific Research with People Act
PHQ-9: Patient Health Questionnaire-9
SD: Standard deviations
